# Association between sports type and overuse injuries of extremities in children and adolescents: a systematic review

**DOI:** 10.1186/s12998-016-0122-y

**Published:** 2016-11-15

**Authors:** Charlène Chéron, Christine Le Scanff, Charlotte Leboeuf-Yde

**Affiliations:** 1 CIAMS, University Paris-Sud, Université Paris-Saclay, F-91405 Orsay Cedex, France; 2 Institut Franco-Européen de Chiropraxie, 72 Chemin de la Flambère, F-31300 Toulouse, France

**Keywords:** Children, Adolescent, Pediatrics, Overuse injuries, Sports type, Extremities, Epidemiology

## Abstract

**Background:**

Sporting activities can cause injuries and overuse injuries of the extremities (OIE) in children have been shown to be more common than injuries caused by trauma. The lower extremity is more frequently affected than the upper extremity in OIE, but it is not known whether injury site and diagnosis vary in different sporting activities.

**Purpose:**

To identify any differences between sports in relation to diagnoses and anatomical areas most likely to be injured.

**Methods:**

A search was made in November 2014 and again in June 2016 in PubMed, SportDiscus, PsycInfo and Web of Sciences. Search terms were: « overuse injuries OR cumulative trauma disorders OR musculoskeletal injuries » AND « extremity OR limb » AND « physical activity OR sport OR risk factor OR predictors OR exercises » AND « child OR adolescent OR young adults ». Inclusion criteria were: 1) prospective, retrospective, or cross-sectional study design; 2) age ≤19 years; 3) the articles must clearly state if reported cases were classified as traumatic or overuse injuries; 4) reporting on OIE in relation to a particular sports type, and 5) sample size >50. A blinded systematic review was conducted.

**Results:**

In all, nine of the 736 identified articles were included, studying soccer, handball, orienteering, running, dance, and gymnastics. The incidence of OIE was given only in a few articles but at least the site and diagnosis of OIE were identifiable. The lower limb is more often affected than the upper in all sports covered, and, in general, the lower leg and knee are the two most often affected areas. However, in handball, the elbow was the second most often reported area, and in gymnastics injuries of the foot appeared to be more frequent than in the other sports. No differences in diagnoses were observed between sports types.

**Conclusion:**

Our work contributes new information, namely that the site of OIE in children and adolescents appears to vary only somewhat between different types of sports. Further well-designed surveillance studies are needed to improve knowledge that can help prevent injuries in children and adolescents participating in sports activities.

## Background

Physical activity is associated with many positive outcomes in children, such as lower risk of obesity [1], cardiovascular benefits [2], and improved cognitive performance [3]. However, it is also a major cause of injuries in children [4], which can lead to significant costs for parents and society [5]. Classically, a sports injury is defined as any physical complaint that is sustained from a sport activity that may or may not result in time loss from sports activities or in a medical consultation [6]. The medical consequences of sports injuries vary, from virtually none to requiring surgery in severe cases. It is possible that physical damages acquired in childhood lay the foundation for future continued or repeated problems extending into adulthood. It is therefore relevant to learn more about the epidemiology of musculoskeletal injuries in childhood.

Classically, sports injuries are further classified as traumatic or overuse injuries. A consensus statement, presented by Fuller et al. in 2006, defined traumatic injury as an “injury resulting from a specific, identifiable event”, whereas an overuse injury was described as one caused by “repetitive micro trauma without a single identifiable event responsible for the injury” [6]. The term “gradual onset” is also often seen in sports literature as a necessary element for the diagnosis of an overuse injury. However, it is not enough that a (painful) condition is defined as an injury merely because the person with the condition (i.e. often pain) happens to participate in a sporting activity, as pain and other complaints may arise also without externally induced injuries. The term “injury” should therefore not be used synonymously with “pain” or “complaint”.

For several reasons the above definitions are not sufficient, as pointed out by Bahr in 2009 [7]. Firstly, the term “event” is not sufficiently clear. Pain may arise “suddenly” following an “event” but it could be the result also of a long-term overuse process, for example in the case of a stress fracture. The sudden onset does not qualify this particular painful complaint to be categorized as a traumatic injury, because it could be the result of long-term overuse without sufficient time for recovery. Therefore, the word “event” should be replaced by “cause”.

Secondly, the sustained injury would depend on the type and duration of activity and the tolerance of the underlying tissues. Bahr discusses the concept that “repetitive low-grade forces exceeding the tolerance of the tissues cause overuse injuries” [7]. This means that the nature and duration of the activities must be considered in relation to the resistance of the anatomical structures of the individual performing these activities.

A systematic review of the US high school sport epidemiology literature was published in 2014 on the definition and usage of the term “overuse injury”. The authors highlighted the fact that some authors consider “overuse” to be a mechanism of injury while others use it as a diagnosis-based definition [8]. When used as a mechanism of injury, “overuse” refers to the causation of the injury, the cumulative or repetitive activity which leads to the injury. When used as a diagnosis, “overuse” often refers to a family of injuries classified by slowly progressing inflammation, pain, and loss of function [8].

Clearly, the definition of an overuse injury is more complicated than that of a traumatic injury. In summary, we suggest that the following aspects need to be respected:

1. There must be a complaint, either simply noted by the player, or in terms of its consequences (e.g. medical attention and/or time-loss).
2. There should be no single identifiable traumatic cause.
3. There should be a history of repeated micro-trauma.
4. It would most likely have a gradual onset but this is not sure.
5. The activities preceding the complaint should be capable of exceeding the tissue tolerance.

Moreover, the nature and area of the complaint should be examined and a diagnosis made by an appropriately trained person.

Musculoskeletal injuries typically affect the back and extremities [9]. Most people probably associate “injury” with obvious trauma, but in a study of a large natural experiment including all children in ten public schools in Denmark aged 6 to 12 years, who were followed weekly by text messages (SMS-track) during 2.5 years [9], overuse injuries of the extremities (OIE) were found to be nearly twice as common as traumatic injuries of the extremities. The most frequent overuse diagnosis of the lower extremity was apophysitis and the upper extremity injuries were most often diagnosed as soft tissue injuries [9].

It is likely that different sports place different physical demands on various parts of the body, and a good understanding of these vulnerable points in different sports should help trainers and performers better to prevent and manage injuries.

We conducted a systematic review to gain a better understanding of sports-specific OIE in children and adolescents. Its purpose was to identify any differences between sports in relation to diagnoses and anatomical areas most likely to be injured.

## Methods

### Systematic literature search

A librarian-assisted search was conducted on November 2014 and a final search performed in June 2016 in PubMed, SportDiscus, PsycInfo and Web of Sciences using the search terms « overuse injuries OR cumulative trauma disorders OR musculoskeletal injuries » AND « extremity OR limb » AND « physical activity OR sport OR risk factor OR predictors OR exercises » AND « child OR adolescent OR young adults » in different combinations (MeSH terms and free text). An additional citation search of reference lists of the retrieved articles was performed. No restrictions were placed on date of publication. No attempts were made to search the grey literature.

### Inclusion criteria

Our approach was modelled on the Preferred Reporting Items for Systematic reviews and Meta-Analysis (PRISMA) flow-chart, as described below [10]. The first author applied the inclusion criteria to the title and abstract of the articles identified from the literature search. Blinded full-text screening was then done by two authors to determine which articles should be included in the final review. Inclusion criteria were: 1) a study design that was prospective, retrospective or cross-sectional, 2) age 19 years or below, 3) the OIE should be reported in relation to a particular sports type, and 4) sample size greater than 50. Only articles written in English, French or a Scandinavian language were considered. A list of articles excluded from the review on the basis of the full-text screening is available from the authors on request.

### Data extraction

Two descriptive checklists, one quality checklist, and three tables of results were created by the authors for the purposes of this review. Information was grouped by sports type.

The first checklist presents information on type of sport, year of publication and country of study, as well as the observational setting (i.e. specific college, sports club or competition), the level of sport, the age and sex of the children, the number of participants and participation rate, the duration of data-collection, and the data source.

The second checklist was used to describe the definitions of injury and overuse injury used in the articles. Regarding criteria used for sports-related injury we chose those described by Fuller [6], whereas our overuse definition was inspired by criteria proposed by Bahr in 2009 [7] which are: repeated micro-trauma, no single identifiable cause, gradual onset, and activity exceeds tissue tolerance. Our classification of overuse injury was based on diagnosis, as described by Roos [8]. Moreover, because some articles did not use the criteria described above we added a column titled “other”, in which we quoted the definition used in the article.

In the third checklist, which related to the methodological quality, we were interested in whether: 1) participation rate was clearly stated, 2) the diagnosis of OIE was collected by medical personnel 3) the incidence of OIE was clearly reported in the article, 4) diagnoses and areas of overuse injury had been clearly and completely reported, and 5) the number of injuries could be reported in relation to the number of hours of exposure.

Checklists 2 and 3 were used to identify the strengths and weaknesses of this research area but were not taken into account during the interpretation of findings.

The findings were reported in three evidence tables. Table 4 shows the incidence of OIE and the proportion of OIE regarding the number of injuries per hour of exposure. Incidence was provided only if explicitly reported in the article. In addition, the proportion of OIE was calculated if information was provided on the number of injuries in relation to the number of hours of exposure.

Table 5 focuses on the site of overuse injuries (e.g. shoulder, elbow). Injuries clearly pertaining to the extremities were included in this table. Diagnoses that were clearly related to a particular site were recorded accordingly (e.g. ‘Osgood Schlatter’ = knee injury). Vague descriptions such as ‘tendinitis’ that could not be linked to a specific anatomical region were not recorded.

Table 6 reported on diagnosis related to injury site. If the diagnosis was provided but not the site, making it impossible to relate it to the limbs, the information was not used in the analysis. In one article on running, the authors reported stress fracture without specifying the localization. We decided to describe these injuries as extremity injuries and to include them in Tables 4, 5, 6 and 6 because we assumed that a stress fracture in running concerned the lower extremity. The two most frequently reported areas or diagnoses were emphasized in the tables.

A MeaSurement Tool for the Assessment of multiple systematic Review (AMSTAR) checklist [11], was used as a guide for this article. However, tests for homogeneity for control for likelihood of publication bias could not be carried out.

### Review process and interpretation of data

The checklists and tables were tested for relevance and user-friendliness, adjusted as needed, and thereafter used by two authors, who separately (and blind to each other’s findings) extracted information from the included articles. The data were later compared to detect extraction errors. If necessary, a third person arbitrated any disagreements between the two reviewers. Interpretation of data was done in collaboration between the first two authors. The review was registered in the PROSPERO database: CRD42014007079.

## Results

### Number of articles

**Fig. 1:**
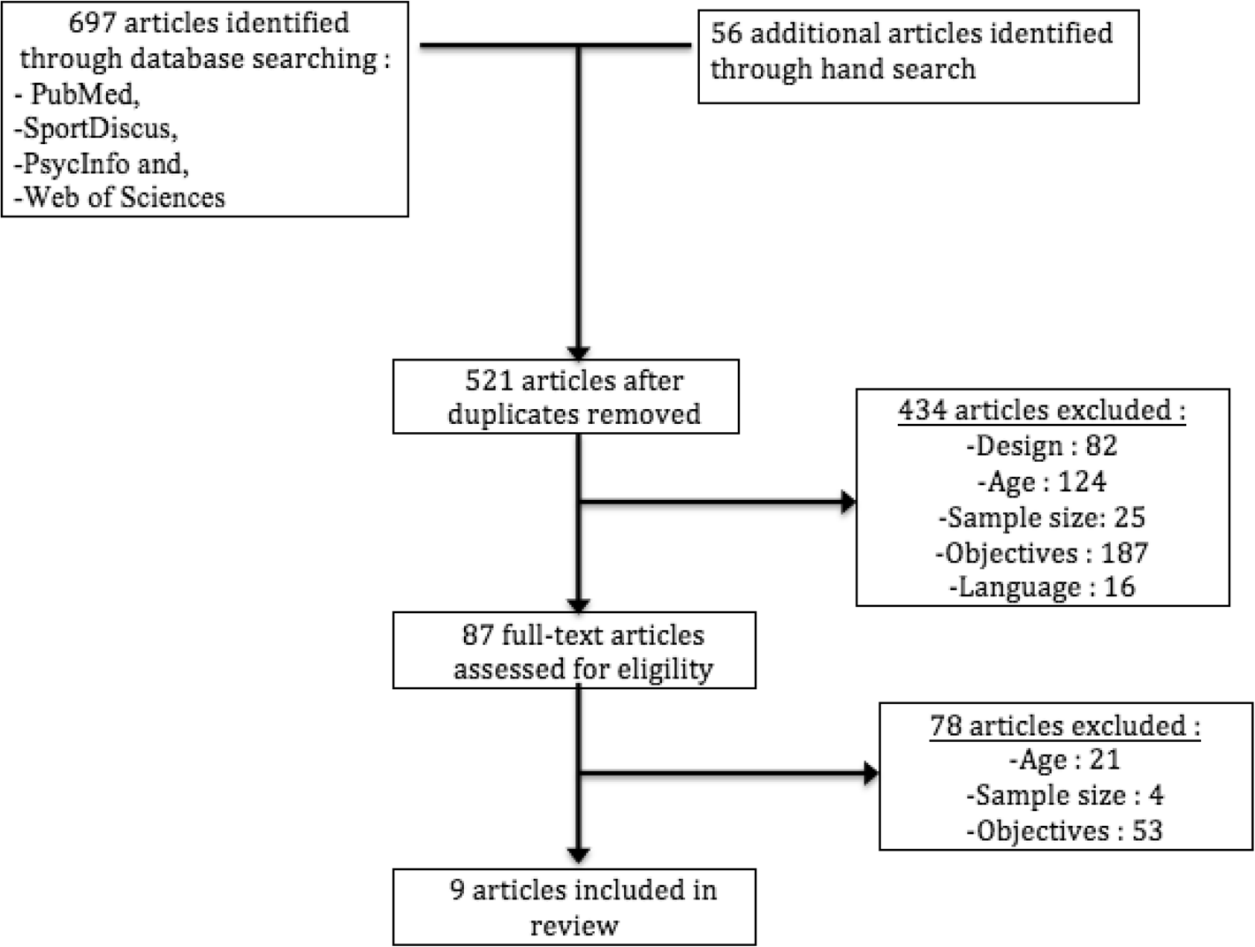
PRISMA flowchart showing selection of articles

After primary selection we obtained 17 articles that fulfilled our initial inclusion criteria out of the 753 articles identified through the database and citation searches (Fig. 1). Most of the excluded studies either did not include children or did not deal with specific sports. However, in some of the 17 articles it was not possible to interpret the data correctly because it was not always clear if a stated condition was an OIE or not. We, therefore, realized that we had to add a new inclusion criterion: “the article must state clearly if reported cases were classified as traumatic or overuse injuries “. With this specific selection we were left with eight articles to which was added one obtained through hand search of reference lists, i.e. a total of nine articles (please see Fig. 1).

### Study design, participants and method of data collection

Seven studies were prospective [12–18] and two were retrospective in design [19, 20]. As can be seen in Table 1, the nine articles included in the analyses reported on 6 different types of sport, i.e. soccer, gymnastics, handball, orienteering, running and dance and were published between 1986 and 2011. Of these, 5 were conducted in Europe, particularly in the Scandinavian countries.

**Table 1:** Characteristics of study participants in nine studies on overuse injuries of the extremities in children and adolescents

Sports	Author year country	Study population level	N part/N invited (participation rate)	Sex	Age Min-max (Mean ± SD)	Duration of data collection & follow-up frequency (N/time)	Method of data collection	Data source
Soccer	Soderman 2001 [12] Sweden	Sport’s club European Level	153/175 (87.4 %)	F	14.1–19.2 (15.9 ± 1.2)	1 season (regular contact)	Physical exam, Injury protocol	Child, Medical personnel, Trainers
	Soligard 2008 [13] Norway	Sport’s club All club	837/1220 (68.6 %)	F	13–17 (?)	1 season (weekly)	Questionnaire	Medical personnel, Trainers
	Le Gall 2008 [14] France	Sport’s club Elite	119/119 (100 %)	F	15–19 (?)	8 seasons (daily)	?	Medical personnel
Handball	Wedderkopp 1999 [17] Denmark	Sport’s club European team	126/? (?)	F	16–18 (?)	1 season (every 10th day)	Physical exam, Questionnaire	Medical personnel, Trainers
Orienteering	Johansson 1986 [18] Sweden	Specific colleges	89/? (?)	B	? (17.5 ± 1.5)	1 season (daily)	Injury protocol, Questionnaire, Interview	Child, Medical personnel, Trainers
Running	Tenforde 2011 [19] USA	High school All club	1196/748 (63 %)	B	13–18 (15.5)	Relates to lifetime	Online questionnaire	Child
Dance	Leanderson 2011 [20] Sweden	Specific colleges	<476/? (?)	B	<10 and 10–14 (?)	7 years (daily)	Physical exam	Medical personnel
Gymnastics	Caine 2003 [15] USA	Sport’s club Different levels	79/? (?)	F	7–18 (?)	3 seasons (bi-weekly)	Physical exam, Injury protocol, Interview	Medical personnel, Trainers
	Lindner 1990 [16] Canada	Sport’s club Different level: provincial to national	178/275 (64.7 %)	F	7–15 (10.61)	3 seasons (regular basis)	Injury protocol, Questionnaire	Child, Trainers

? : Information not provided

*F* female, *B* both

As can be seen in Table 1, the number of children and adolescents observed in the various studies ranged from 79 to 837, with response rates varying from 63 to 100 %. Three of the articles included children and adolescents (ranging between 7 and 18 years), while six articles included only adolescents (between 12 and 19 years). Six studies included only girls, and the other three studies included both boys and girls. Duration of data collection varied from one season to eight seasons but was not always well described. Moreover, the data source was either the child, the trainer, or medical personnel or, usually, a combination thereof.

### Definitions of injury and overuse injury

Table 2 shows how the definition of “injury” differed between articles. In general, the definition was tied to complaints related to sport activities concerning their consequences (i.e. time loss or medical attention). The same table shows how the overuse aspect was not well defined and quite heterogeneous. None took into account whether the activity exceeded the tolerance of tissue, repeated micro trauma was only included in one article, and only two used the criterion of no single identifiable cause, whereas gradual onset was commonly used as the only criterion of overuse injury. Three articles did not use any of these criteria. In one article, only the title indicated the topic. In another one, an example of overuse injury was given, and in a third article, the diagnosis seemed to be one of exclusion (if not traumatic in origin then it had to be overuse).

**Table 2:** Criteria used to define injury and overuse injuries in nine studies on overuse injuries of the extremities in children and adolescents

Sports	Authors year	Criteria for injury				Criteria for classified injury as overuse				
		Sport-related	Complaint	Time-loss	Medical attention	Repeated microtrauma	No single, identifiable cause	Activity exceeds tissus tolerance	Gradual onset	Other
Soccer	Soderman 2001 [12]	X		X			X		X	
	Soligard 2008 [13]	X		X			X		X	
	Le Gall 2008 [14]	X	X	X		X				
Handball	Wedderkopp 1999 [17]	X		X						Classified traumatic or and overuse injury from the diagnosis: for example tendinitis being overuse injury and a sprain being an acute traumatic injury.
Orienteering	Johansson 1986 [18]	X		X					X	
Running	Tenforde 2011 [19]									No definition at all but they called their injury “overuse injuries”
Dance	Leanderson 2011 [20]				X					“Traumatic injuries in case the pain result of a defined trauma. All other injuries were deemed to be caused by overuse.
Gymnastics	Caine 2003 [15]	X		X					X	
	Lindner 1990 [16]	X	X	X					X	

### Quality of the studies

In five of the articles, the study response rate was not reported and could not be calculated (Table 3). The diagnosis was established by health personnel in six of the nine studies.

**Table 3:**
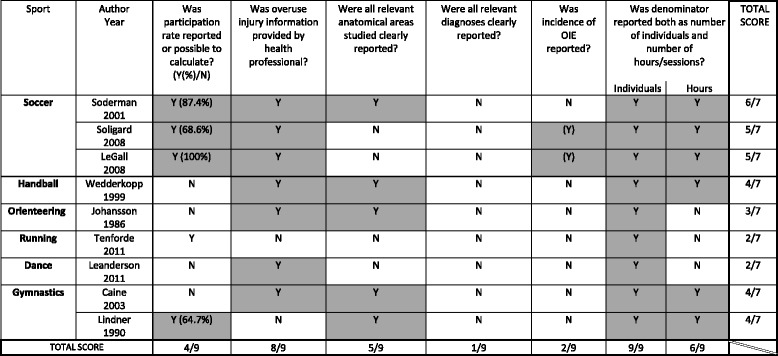
Quality checklist of methodological aspects of nine studies on overuse injuries of the extremities (OIE) in children and adolescents

*N* No

*Y* Yes; when positive answers have been highlighted

Specific sites and diagnoses of OIE were often incompletely reported. Only two articles showed the incidence of some of the OIE. Nevertheless, proportions of injuries in terms of hours of exposure could be calculated in six articles.

### Incidence estimates and proportions of overuse injuries

Table 4 shows the results regarding incidence and proportions of OIE. Specific incidence estimates were available only in two articles on soccer, for the diagnoses periostitis, tendon pain, and tendinopathy, at <0.5 OIE for 1000 h of exposure.

**Table 4:** Incidence and proportion of overuse injuries of the extremities (OIE) based on numbers of hours of exposure in nine studies on children and adolescents

Sport	Author year	Number of OIE	Incidence given in the article	Number of hours of exposure	Proportions of OIE based on number of hours of exposure (1000)
Soccer	Soderman 2001 [12]	21	-	Per player: -Training: 46.9 ± 17.1 -Game: 29.5 ± 14.8	1.80
	Soligard 2008 [13]	33 of which: -Lower extremity tendon pain: 21 -Anterior leg pain: 12	Yes for: -lower extremity tendon pain: 0.5 -anterior leg pain: 0.3	Total: 45428 of which: -Training: 31086 -Game: 14342	Total: 0.73 of which: -Lower extremity tendon pain 0.46 -Anterior leg pain: 0.26
	Le Gall 2008 [14]	Only for tendinopathy: 35	Only for tendinopathy: 0.36	Total: 97325 of which: -Training: 87530 - Game: 9795	For tendinopathy: 0.36
Handball	Wedderkopp 1999 [17]	8	-	Total: 17945	0.04
Orienteering	Johansson 1986 [18]	38	-	-	-
Running	Tenforde 2011 [19]	822	-	-	-
Dance	Leanderson 2011 [20]	127	-	-	-
Gymnastics	Caine 2003 [15]	55	-	Total: 76919.5	0.72
	Lindner 1990 [16]	17	-	Total: 173263	0.10

The highest overall proportion of OIE was found for soccer: 1.8/1000 h of exposure, followed by 0.7 in one study on soccer, and in one study on gymnastics. A third study on soccer had an overall proportion of OIE of 0.4/1000 h of exposure, whereas handball and another study on gymnastics had lower estimates (<=0.1/1000 h of exposure).

### Injury site and diagnosis in general

In general, the lower limb was most often affected, and especially the knee, tibia, and thigh (Table 5). The most frequently provided diagnoses were tendinitis/bursitis and periostitis (Table 6).

**Table 5:**
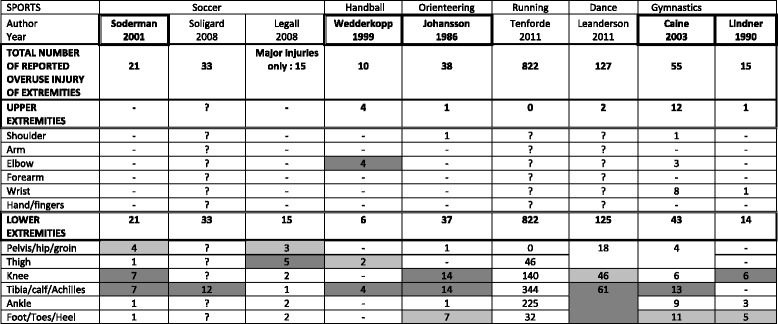
Site of overuse injuries of the extremities by sports type in nine studies on children and adolescents

“?” = Information not provided

The two most common injury sites in each article are highlighted:  for the most common and  for the second most common

Articles in which all OIE are described and in which all the sites of OIE are clearly described are framed i.e.

**Table 6:**
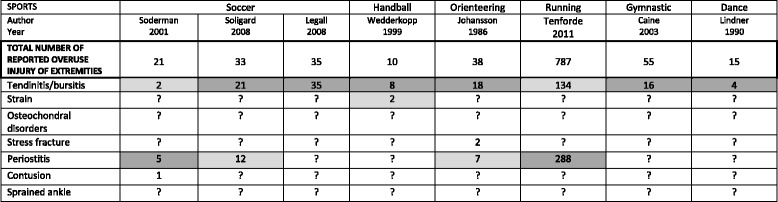
Injury diagnosis according to sports type for eight studies on children and adolescents that included specific diagnosis

The two most common diagnoses in each article are highlighted:  for the most common and  for the second most common

Articles in which all OIE are described and in which the diagnoses are clearly described are framed i.e.

Leanderson did not mention any diagnosis so we did not included it on this table

### Differences in overuse injuries according to sports type

For all sports covered, the lower limb was more often affected than the upper. It was not possible to compare the incidence rates because it was only rarely provided. In handball, elbow injuries were equally common and noted as the most common injury site equal to the lower leg injuries. The foot was the second most common site in gymnastics. No differences in diagnosis could be found between different types of sports, mainly because only few articles clearly provided the diagnosis.

## Discussion

### Summary of results

To our knowledge, this is the first systematic review on this topic. Our aim was to improve the understanding of the relationship between type of sport and OIE in children and adolescents. The individuals included in this study were more often adolescents than children, but the articles included did not allow us to distinguish between these two groups. Whilst the reporting styles of the reviewed articles made it impossible to compare exact incidence rates, relative differences in occurrence could be studied in relation to the proportion of injuries per numbers of hours of exposure and injury site and diagnosis.

In a previous large study on 1259 school-children aged from 6 to 12 years old, in which data were collected with weekly text messages over 2.5 years, the lower extremities were generally affected more often than the upper extremities [9]. The results of our review largely confirm this finding. Specifically, our results indicate that the most commonly injured anatomical sites were the knee and lower leg.

They were two exceptions to this: handball and gymnastics. In handball the most commonly injured areas were elbow and the lower leg; they were equally frequent. In the two articles concerning gymnastics, the foot was the second most common affected area. In one article, the knee was in the first position, whereas in the other it was the lower leg.

Furthermore, our results indicated the most common diagnoses to be tendinitis/bursitis and periostitis and these were similar across all sports (when reported) but, again, no incidence estimates could be extracted.

### Methodological aspects of the articles reviewed

In relation to the external validity of these results, it is not known how representative the study samples would be for children as a whole, as the articles reported on specific sport groups and there were no population-based studies. The main objective in most of the studies was to describe the musculoskeletal problems that occurred in a sports club or school, i.e. using convenience samples. A larger number of studies would probably have given a wider range of results. Low response rate could also affect the validity of data, and response rate information was provided in only five studies.

Injury was never clearly defined on its own. Instead, problems arising in relation to a sport activity would be reported if there was also a time loss for that sport, or if medical attention was required. The definition of “injury” was therefore a definition of consequences, e.g. time-loss and/or medical attention, rather than a painful condition.

Further, the overuse definition lacked a substantial pathological aspect relying mainly on the observation of gradual onset of symptoms. Bahr has already discussed this problem [7]. We propose that it would probably decrease the risk for misclassification, if at least three of the following criteria were fulfilled: repeated micro trauma, no single identifiable cause, activity exceeds tissue tolerance, and gradual onset.

Furthermore, the level of expertise differed between persons responsible for defining the injury, ranging from medical specialist to the injured individual. Depending on the definition and site of injury, it can be expected that self-reported problems might be less accurate than those obtained through a medical examination. The definition is important when comparing estimates between studies, because, as pointed out by Bahr, self-reported pain will result in higher estimates than pain with a consequence in terms of medical attention or time-loss [7].

The fact that not all diagnoses and sites of injuries were clearly reported in each article was an additional difficulty.

In sum, this area of research would benefit from a well-reasoned consensus approach to the various relevant definitions, making it possible to compare findings between studies.

### Methodological aspects of our review

Relevant articles written in other languages than those included in our review may exist and their inclusion could have changed the results.

Checklists and evidence tables used for data extraction were tested for user-friendliness and adjusted in a pilot study before being used in the main study. The review was carried out independently by two reviewers, one of whom was experienced in performing systematic reviews. Because the objectives of the articles were different from ours, it was frequently necessary to discuss and interpret their texts to correlate with our checklist but the third reviewer never had to be called in for arbitration.

### Discussion of findings regarding the anatomical site of OIE

In some sports, such as handball and gymnastics, the upper limb is subjected to more repetitive stress than in other sports, such as soccer and running. One could assume that it therefore should be affected at least as often as the lower limb but, generally even in these sports the lower limb was most commonly affected. Perhaps the explanation for this is that the lower limb always carries the weight of the body, which puts the lower limb in a constant stress situation. In comparison with this constant weight, other sport-associated demands on the upper body are probably much less important.

### Discussion of findings regarding the diagnostics of OIE

The results of our study indicate that the most frequent diagnoses are fairly similar across sports, mainly reported as tendinitis/bursitis, and periostitis. Not surprisingly, OIE seem to occur mainly where the bodily structures are most subjected to repeated stress.

## Conclusion

Paucity of relevant articles and lack of information in those we found prevented us from defining and comparing the incidence of OIE between sports for children and adolescents. Our research highlights the fact that further and better-detailed studies are required to obtain useful information on OIE and its prevalence in different types of sport. This may lead to improved training programs, prevention and management of OIE. Nevertheless, this review provides some useful information on OIE on children and adolescents. Whilst, not unexpectedly, the site of OIE varies between different types of sport, we find that the lower limb is more often affected than the upper limb irrespective of the type of sport.

## Abbreviation

OIE: Overuse injuries of extremities
